# Data assimilation using a global Girsanov nudged particle filter

**DOI:** 10.1007/s11222-026-11019-z

**Published:** 2026-11-01

**Authors:** Maneesh Kumar Singh, Joshua Hope-Collins, Colin J. Cotter, Dan Crisan

**Affiliations:** 1 Department of Mathematics, Imperial College London, London, UK; 2 Department of Mathematics, SRM Institute of Science and Technology, Kattankulathur, Chennai India

**Keywords:** Particle filter, Data assimilation, Girsanov theorem, Stochastic partial differential equations

## Abstract

We present a particle filtering algorithm for stochastic models on infinite dimensional state space, making use of Girsanov perturbations to nudge the ensemble of particles into regions of higher likelihood. We argue that the optimal control problem needs to couple control variables for all of the particles to maintain an ensemble with good effective sample size (ESS). We provide an optimisation formulation that separates the problem into three stages, separating the nonlinearity in the ESS term in the functional with the nonlinearity due to the forward problem, and allowing independent parallel computation for each particle when calculations are performed over control variable space. The particle filter is applied to the stochastic Kuramoto-Sivashinsky equation, and compared with the temper-jitter particle filter approach. We observe that whilst the nudging filter produces an ensemble with a larger spread than the temper-jitter filter, it responds to extreme events in the assimilated data more quickly and robustly.

## Introduction

Data assimilation can be defined as the blending of observational data and computational models to obtain estimates of the past, present and future state of a physical system. It is a critical tool in weather and climate which is now spreading across the engineering, physical and biological sciences (Reich and Cotter 2015; Nakamura and Potthast 2015; Law et al. 2015; Carrassi et al. 2018). In this article we focus on the filtering problem, which seeks the Bayesian estimate of the probability distribution for the model solution at time $t=t_0$, conditioned on noisy observations at times $0<t\le t_0$ and a prior distribution at time t=0, and its solution by sequential methods that incrementally increase $t_0$. This distribution can be used to quantify uncertainty about the current state as well as the future state by forward propagation using the computational model, to produce a forecast. The gold standard for approximate computational solution of the filtering problem is the particle filter, which evolves an empirical approximation of the filtering distribution using an ensemble of model states that are propagated in time using the computational model and then conditioned on the observed data by weighting and resampling. For the classical bootstrap particle filter, this ensemble needs to be large for useful estimates. A summary of analytical results for particle filters is given in Crisan and Doucet (2002).

When the model state space is large, as arising from the discretisation of a (possibly stochastic) partial differential equation (PDE), having a large number of particles becomes impractical, and modifications and compromises are sought. In operational systems, the ensemble Kalman filter is a highly successful approach, based on approximating the distributions as multivariate normal and calculating a transformation of the ensemble of states instead of weighting and resampling (Evensen 2009). These are combined with localisation techniques to avoid finite size effects causing spurious long range correlations (Houtekamer and Mitchell 1998; Hunt et al. 2007). However, there has also been a number of efforts to adapt particle filters to the smaller ensemble setting, such as equal weights particle filters (Ades and Van Leeuwen 2015; Zhu et al. 2016), filters with Gaussian resampling (Potthast et al. 2019), filters with “tempering” and “jittering” (Beskos et al. 2017; Cotter et al. 2020b, a), and filters with localisation (Poterjoy 2016; Crisan and Fausti 2025). The goal of these efforts is to avoid assumptions about the distribution that underpin the ensemble Kalman filter.

In this article we focus on the incorporation of a “nudging” procedure into particle filters. Nudging is a long established technique in data assimilation (Kalnay 2003) that simply adds a relaxation term that “steers” the solution towards the observed data. More recently, there has been a programme of rigorous analysis to prove convergence of variants of these nudging schemes in the large time limit (Olson and Titi 2003; Azouani et al. 2014; Bessaih et al. 2015). In particle filters, the goal of nudging is to move the ensemble of states such that their likelihood is increased, without losing consistency of the filter asymptotically in the limit of many particles. One approach is to modify the proposal (Chorin et al. 2010; Zhu et al. 2016). In another approach, it was shown in Akyildiz and Míguez (2020), González et al. (2025) that ensemble members can simply be nudged in directions of increasing likelihood which introduces bias but still gives asymptotic convergence for large ensembles provided that certain assumptions are met. Nudging can also be used to try to keep the particle weights more uniform, to avoid "weight collapse" where almost all the weight is assigned to just a few particles. In the feedback particle filter (Yang et al. 2013), nudging terms are introduced and an optimal control formulation keeps all of the weights exactly equal, leading to a PDE to be solved over the optimal control state space. Abedi et al. (2022) proposed a generalisation of this idea to the McKean-Vlasov setting, in which the particle weights have their own dynamics that can then be controlled to avoid weight collapse.

In this article, we take a different approach based on a Girsanov modification of a stochastic (partial) differential equation (S(P)DE). In this setting, the modification adds deterministic control variables to the S(P)DE with a corresponding reweighting specified by Girsanov’s Theorem. We are free to choose these variables provided that they do not depend on the forward filtration of the stochastic process. This means that they can only depend on the noise realisations for times in the past. Instead of solving a high-dimensional PDE for the optimal controller, we solve a pathwise optimisation problem restricted to the assimilation window. In previous work (Cotter et al. 2020a, 2025), we independently optimised the control variables to maximise the Girsanov adjusted particle weights, independently for each particle. This turns out not to improve the filter performance, because although all of the particles move closer to the region of high probability, there are still a few particles that have almost all of the weight. In this work, we take a different approach, by solving a global optimisation problem defined on all the control variables of all of the particles together. The functional has two terms, one encourages the ensemble towards regions of high Girsanov-modified likelihood, whilst the other term ensures that the ensemble members are roughly equally weighted. This can be viewed as a multiobjective optimisation problem. Solving this problem couples together the control variables for all of the ensemble members; the landscape of the functional makes a direct approach intractable for nonlinear SPDEs. In this article, we address this challenge by providing an approximate solution of this problem that breaks it down into three stages. The first and third stages solve uncoupled problems for each ensemble member, which can be solved in parallel. These problems depend on the forward solution of the S(P)DE, and their practical solution is enabled by automated adjoint computation. The second stage connects all of the ensemble members together, but only requires the solution of a constrained optimisation problem in $\mathbb {R}^N$, where *N* is the ensemble size, and without requiring forward/adjoint model solutions. Hence, the algorithm is feasible in the high performance computing setting.

The main contributions of this article are:

1. The idea of solving an optimisation problem for the global set of Girsanov control variables for the whole ensemble of particles.
2. The formulation of a new three stage algorithm that decouples the high dimensional component of the optimisation, namely the structure of the control variables, so that each particle can optimise its own control variables independently (and in parallel). The only part of the optimisation that is global is over the Girsanov adjusted weights themselves, i.e. an $N_p$ dimensional nonlinear problem where $N_p$ is the number of particles.
3. The investigation of particle filters using this new algorithm using the stochastic Kuramoto-Sivashinsky equation.

The rest of this paper is organised as follows. In Sect. 2 we establish some notation and preliminaries before describing our nudging framework and the three stage solution approach. In Sect. 3 we provide some numerical experiments illustrating our approach, and in Sect. 4 we provide a summary and outlook.

## Girsanov nudged particle filtering

In this section, the details of our particle filtering approach will be presented.

### Notation and preliminaries

We adopt the stochastic nonlinear filtering framework for data assimilation. Let (*X*, *Y*) be processes on $(\Omega , F, \mathbb {P})$, where *X* is the (hidden) signal and *Y* is a discrete time observation process

1 $$\begin{aligned} Y_{t_k} := h(X_{t_k}) + V_{t_k}, \end{aligned}$$

where $h: \Omega \rightarrow \mathbb {R}^M$ is the observation operator, $V_t$ is a discrete time noise process, and $(t_1,t_2,t_3,\ldots )$ is a sequence of observation times. The probability density function $L(Y_{t_k}|X_{t_k})$ for $Y_{t_k}$ conditional on the value of $X_{t_k}$ is referred to as the likelihood. We will work with the negative log likelihood $\Phi (X_{t_k}, Y_{t_k})=-\log L (Y_{t_k}|X_{t_k})$.

Here, *X* solves a given SPDE, and the goal is to estimate the filtering distribution $\pi _{t_k}(X_{t_k}|Y_{t_1},Y_{t_2},\ldots ,Y_{t_k})$ for the signal at $t=t_k$ given all observations $Y_{t_j}$ for j=1,…,k. Bayes’ rule states that

2 $$\begin{aligned} \frac{{{\,\textrm{d}\,}}\pi _{t_k}(X_{t_k}|Y_{t_1},Y_{t_2},\ldots ,Y_{t_{k-1}},Y_{t_k})}{{{\,\textrm{d}\,}}\pi _{t_k}(X_{t_k}|Y_{t_1},Y_{t_2},\ldots ,Y_{t_{k-1}})} = L(Y_{t_k}|X_{t_k}), \end{aligned}$$

where ${{\,\textrm{d}\,}}\pi _A/{{\,\textrm{d}\,}}\pi _B$ is the Radon-Nikodym derivative of $\pi _A$ with respect to $\pi _B$.

We approximate $\pi _t$ using *particle filters*, which are sequential Monte Carlo methods representing the conditional law of $X_t$ via a weighted ensemble of model states, or particles, $\{(w_i,X_i)\}_{i=1}^{N_p}$, with

$$ \pi _{t} \approx \sum _{i=1}^{N_p} w_i\delta (X-X_i(t)). $$

In the most basic bootstrap particle filter, particles are propagated forward in time from $t=t_{k-1}$ to $t=t_k$ using independent realisations of the stochastic process modelling the state for each particle. Then, each particle $X_{i,t}$ is assigned an updated likelihood weight *via*

$$ w_i \mapsto \hat{w}_i = w_i L(X_{i,t_k}|Y_{t_k}), $$

which are then normalised according to $w_i=\hat{w}_i/\sum _{j=1}^{N_p}\hat{w}_j$.

To assess weight degeneracy, we compute the *effective sample size* (${{\,\textrm{ESS}\,}}$),

3 $$\begin{aligned} {{\,\textrm{ESS}\,}}({\textbf{w}}) {:=}\left( \sum _{i=1}^{N_p} {w}_i^2 \right) ^{-1}{,} \end{aligned}$$

where $\textbf{w}=(w_1,w_2,\ldots ,w_{N_p})$. Here, ${{\,\textrm{ESS}\,}}\approx N_p$ implies uniform weights, while ${{\,\textrm{ESS}\,}}\ll N_p$ indicates a loss of ensemble spread which will lead to filter divergence. Resampling is triggered when the normalised ESS, $N_p^* = {{\,\textrm{ESS}\,}}/N_p$, falls below a threshold, resulting in a new set of equally weighted particles which are sampled from the original set with replacement in such a way that the empirical distributions before and after resampling are asymptotically consistent as $N_p\rightarrow \infty$. This has the effect of replicating higher weight particles and discarding lower weight particles (on average). In this article we used the systematic resampling algorithm of Gordon et al. (1993).

### The Girsanov nudging framework

The effective sample size reflects weight degeneracy, and we frequently observe it dropping in high dimensions (such as discretisations of SPDEs) due to particle sparsity in observation space. This leads to poor posterior approximation.

In this framework, we introduce a time-dependent *control variable* to guide particles toward regions suggested by observations. This *nudging* preserves consistency, meaning the particle ensemble remains a valid set of samples from the prior distribution after appropriate weight modification.

We justify this approach through a stochastic differential equation (SDE),

4 $$\begin{aligned} {{{\,\textrm{d}\,}}X_t = f(X_t){{\,\textrm{d}\,}}t + \sigma (X_t){{\,\textrm{d}\,}}W_t, \quad \mathcal {L}(X_0) = \pi _0 }, \end{aligned}$$

where $X \in \mathbb {R}^{N_1}$, $f:\mathbb {R}^{N_1}\rightarrow \mathbb {R}^{N_1}$, $\sigma : \mathbb {R}^{N_1}\rightarrow \mathbb {R}^{N_1 \times N_2}$, *W*(*t*) is a $N_2$-dimensional Brownian motion, and $\pi _0$ is a probability measure describing the distribution of the initial condition at time 0. In a bootstrap particle filter, we consider a set of $N_p$ particles $\{X_{i,t}\}_{i=1}^{N_p}$ with initial conditions $X_{i,0}$ drawn from $\pi _0$, each solving (4). The empirical distribution given by

5 $$\begin{aligned} \pi _{T,N_p} = \frac{1}{N_p}\sum _{i=1}^{N_p}\delta _{X_{i,T}} \end{aligned}$$

provides an approximation of the prior distribution $\pi _T$ for $X_t$ at time *T* (before taking the observation). At time *T*, each particle is given a weight as described in the previous section, to approximate the posterior distribution by the empirical distribution,

6 $$\begin{aligned} \pi _{T,N_p|y} = \sum _{i=1}^{N_p}w_i\delta _{X_{i,T}}. \end{aligned}$$

This is then iterated for further assimilation windows.

If we instead solve a modified SDE,

7 $$\begin{aligned} {{\,\textrm{d}\,}}\hat{X} = f(\hat{X}){{\,\textrm{d}\,}}t + \sigma (\hat{X})\left( \lambda (t){{\,\textrm{d}\,}}t + {{\,\textrm{d}\,}}W\right) , \quad \hat{X}(0) = X_0, \end{aligned}$$

where $\lambda (t) \in \mathbb {R}^{N_2}$, the joint probability measure for $\hat{X}(t)$ is absolutely continuous with respect to that of *X*(*t*). The Radon-Nikodym derivative (or Girsanov factor) between these measures is given by

8 $$\begin{aligned} G(\lambda ) = \exp \left( -\int _0^T \frac{1}{2}|\lambda (t)|^2 {{\,\textrm{d}\,}}t \,+ \int _0^T \lambda (t)\cdot {{\,\textrm{d}\,}}W\right) , \end{aligned}$$

subject to Novikov’s conditions on $X_t$ (see Corollary 5.13 of Karatzas and Shreve 1998, for example), and subject to the condition that λ(t) does not depend on the future increment W(s)-W(t) for s>t. Given choices $\{\lambda _i(t)\}$ for each particle $\hat{X}_{i,t}$, $i=1,\ldots ,N_p$, we obtain solutions $\hat{X}_{i,t}$ that depend on $\lambda _i$ through (8), and get new empirical approximations of the posterior distribution at time *T*,

9 $$\begin{aligned} \hat{\pi }_{T,N_p,\Lambda |y} = \sum _{i=1}^{N_p}\tilde{w}_i\delta _{\hat{X}_{i,T}} \end{aligned}$$

where $$\Lambda =(\lambda _1,\ldots ,\lambda _{N_p})$$ and

10 $$\begin{aligned} \tilde{w}_i = \frac{L(X_{i,T}|y)G(\lambda _i)}{\sum _{j=1}^{N_p} L(X_{j,T}|y)G(\lambda _j)}, \end{aligned}$$

whose consistency as $N_p\rightarrow \infty$ is assured by Girsanov’s theorem.

Thus, we can choose λ to steer the particle *X* towards regions of higher likelihood function and/or to achieve more equal weights, but we must compensate by multiplying the weight by *G*. In other words, we pay a cost for nonzero λ(t).

In the discrete time setting, we need to approximate the time integrals in (8) by quadrature sums, e.g.,

11 $$\begin{aligned} G \approx G_{\Delta t} = \exp \left( -\sum _{n=1}^{N_s}\left( \frac{1}{2} |\lambda ^n|^2 \Delta t - \lambda ^n \cdot \Delta W^n\right) \right) , \end{aligned}$$

where $\Delta W^n$ are suitable independent Brownian increments and Δt is the time step size.

In the context of particle filtering, this means that we can solve (7) instead of (4) to propagate each particle from $t_{k-1}$ to $t_{k}$, but now the weight must be updated using the Girsanov-adjusted formula,

$$ w_i \mapsto \hat{w}_i = w_i \exp (-\hat{\Phi }_i), $$

where

12 $$\begin{aligned} \hat{\Phi }_i= &  \Phi (X_{i,t_k},Y_{t_k})+\int _{t_{k-1}}^{t_k} \frac{1}{2}|\lambda _i(t)|^2 {{\,\textrm{d}\,}}t \nonumber \\ &  \quad - \int _{t_{k-1}}^{t_k} \lambda _i(t)\cdot {{\,\textrm{d}\,}}W_i, \quad \nonumber \\ i= &  1,\ldots , N_p. \end{aligned}$$

Here we can select different controls $\lambda _i$ for each particle $X_i$. The Girsanov nudged particle filter is consistent with the original filtering problem, since it does not introduce additional bias in the target distribution. While the particle dynamics are modified by introducing the control term λ(t), this modification is exactly compensated by a corresponding weight correction. So the weighted particle system still targets the correct posterior distribution. In other words, although the proposal dynamics are altered to guide particles toward regions of higher likelihood, the importance weights ensure that this change of proposal does not bias the estimator. This is analogous to standard importance sampling: the proposal distribution is changed to improve efficiency, but the weights correct for this change so that expectations remain unchanged. Therefore, the method retains asymptotic consistency as the ensemble size increases.

This leads naturally to the idea of choosing $\lambda _i(t)$ such that $\hat{\Phi }_i$ is increased where possible whilst avoiding weight collapse. However, it is essential to ensure that Girsanov’s theorem remains valid. This requires that the control λ(t) depends only on the past values of the noise process, i.e., on *W*(*s*) for s<t. To satisfy this adaptivity constraint, we adopt a sequential optimisation strategy. The idea is to construct λ(t) incrementally as we uncover new values of *W*(*t*). Upon discretising time, and defining $\lambda _{i}(t_{k-1} + n\Delta t) = \sum _{j=1}^n \Delta \lambda _{i,j}, i=1,2,\ldots ,N_p$ and $n=1,2,\ldots ,N_s$ such that $t_k=t_{k-1}+N_s\Delta t$, we proceed as follows. First, initialise $\Delta \lambda _{i,n} = 0$ and $\Delta W_{i,n} = 0$ for $n = 1, 2,\ldots , N_s$, and $i=1,\ldots ,N_p$. Then we sample $\Delta W_{i,1}$ from the appropriate distribution, and adjust $\Delta \lambda _{i,1}$ to decrease $\hat{\Phi }_i$, when computed by solving the forward model for $X_i$, with all other parameters fixed, for $i=1,2,\ldots ,N_p$. Then we sample $\Delta W_{i,2}$ and adjust $\Delta \lambda _{i,2}$ and so on, until we reach $n=N_s$.

### Three stage optimisation process

In previous work (Cotter et al. 2020a, 2025) we have investigated the application of this idea to particle filtering for SPDEs. The value of $\lambda _i$ was chosen to minimise $\hat{\Phi }_i$, thus maximising the Girsanov-adjusted weight. This amounts to resolving a trade-off between maximising the likelihood and minimising the Girsanov “cost”. Cotter et al. (2020a) made an initial investigation where $\lambda _i$ was only nonzero for the final stage of the final timestep before the assimilation time $t_k$. This restriction means that $\lambda _i$ can be obtained as the solution of a least squares problem. However, this provides limited opportunity to steer the solution between assimilation times, only providing an impulsive kick right at the end. Cotter et al. (2025) implemented a more flexible optimisation framework where $\hat{\Phi }_{i}$ is optimised for more general time dependencies in $\lambda _i$ (stepwise constant values in this case), along with a parallel implementation where the optimisation problems are solved independently in parallel. These optimisation problems are solved using a gradient method, with the gradient being computed *via* the adjoint equations. In that work, we established that this approach can produce consistent filtering solutions, but it required combination with additional tempering and jittering steps as introduced in Beskos et al. (2017), Cotter et al. (2020b) to obtain stable results. This is because independent optimisation of $\hat{\Phi }_i$ for each particle $X_i$ does not address the problem of filter divergence. The frequent failure of the bootstrap filter in high dimensions is because a small number of particles will acquire almost all of the normalised weight, because they have larger likelihoods than the others and the likelihood function is typically exponentially decaying. This is observable in low ${{\,\textrm{ESS}\,}}$ values, and results in almost all particles having the same state after resampling. If we individually optimise each $\hat{\Phi }_i$, although all particles are moved to regions of higher likelihood, the particles that would have otherwise been close can be moved even closer, and the ${{\,\textrm{ESS}\,}}$ remains low.

To try to keep ${{\,\textrm{ESS}\,}}$ as high as possible, in this article we take a different approach and propose to solve a global optimisation problem over all particles, i.e. over the full set $(\lambda _1,\lambda _2,\ldots ,\lambda _{N_p})$ of control variables. This is closely related to the idea behind the equal weights particle filter (Zhu et al. 2016). Here, we consider minimising the functional

13 $$\begin{aligned} {\mathbb {F}(\hat{\Phi }_1,\ldots ,\hat{\Phi }_{N_p}) = \frac{-\left( \sum _{j=1}^{N_p} \exp (-\hat{\Phi }_j)\right) ^2}{\sum _{k=1}^{N_p} \exp (-2\hat{\Phi }_k)}}, \end{aligned}$$

which reduces to minus the usual ${{\,\textrm{ESS}\,}}$ formula upon setting $\hat{w}_i=\exp (-\hat{\Phi }_i)$ and applying normalisation. (Note that care must be taken here to avoid issues with numerical underflow.) Due to the normalisation factor, this optimisation problem is ill-posed, since scaling all the weights by a constant will not change the ${{\,\textrm{ESS}\,}}$. To deal with this we add a regularisation term, minimising

14 $$\begin{aligned} {\mathbb {F}_{\sigma }(\hat{\Phi }_1,\ldots ,\hat{\Phi }_{N_p})} = \sigma {\left( \sum _{i=1}^{N_p} \hat{\Phi }_i\right) } - {\dfrac{\left( \sum _{j=1}^{N_p}\exp (-\hat{\Phi }_j)\right) ^2}{\sum _{k=1}^{N_p} \exp (-2\hat{\Phi }_k)}}, \end{aligned}$$

where σ>0 is a chosen penalty coefficient. This modification will tend to move particles into regions of Girsanov adjusted weight if it is possible without unduly reducing ${{\,\textrm{ESS}\,}}$.

In our experiments, optimisation algorithms for this problem converged very slowly, probably due to the composition of the nonlinearity of the forward model producing $\hat{\Phi }_i$ with the nonlinearity in the ${{\,\textrm{ESS}\,}}$. This problem also requires an additional layer of parallel communication between particles which are otherwise running independently. To circumvent this, we designed the following three stage algorithm to find approximate solutions of this optimisation problem. In the first stage, we find $\lambda _i$ that minimises $\hat{\Phi }_i$ separately for each particle $X_i$. This can be done for each particle independently in parallel. The result determines the possible ranges of values $\hat{\Phi }_{i,\min }$ (from the optimising $\lambda _i=\lambda _i^*$) and $\hat{\Phi }_{i,\max }$ (from $\lambda _i=0$) for $\hat{\Phi }_i$ in the global optimisation. In the second stage, we minimise $\mathbb {F}_{\sigma }$ in (14) over $(\hat{\Phi }_1,\hat{\Phi }_2,\ldots ,\hat{\Phi }_{N_p})$ instead of over $(\lambda _1,\lambda _2,\ldots ,\lambda _{N_p})$, subject to the constraints

$$ \hat{\Phi }_{i,\min } \le \hat{\Phi }_{i} \le \hat{\Phi }_{i,\max }, \quad i=1,2,\ldots ,N_p. $$

This is a problem in state space dimension $N_p$, and does not involve running the forward model. After computing the optimal values $(\hat{\Phi }^*_1,\hat{\Phi }^*_2,\ldots ,\hat{\Phi }^*_{N_p})$ in stage two, in stage three we need to find the value of $\lambda _i$ that produces $\hat{\Phi }_i=\hat{\Phi }^*_i$ for $i=1,2,\ldots ,N_p$. There are multiple solutions to this problem, but we set $\lambda _i=s_i\lambda _i^*$ for $s_i\in \mathbb {R}$, and seek the value of $s_i$ that achieves the optimal $\hat{\Phi }^*_i$, which is reachable provided that $\hat{\Phi }_i$ is continuous when viewed as a function of $s_i$. This is a one-dimensional nonlinear equation that can also be solved independently in parallel for each particle $X_i$.

To state the algorithm more precisely, we assume that the SPDE is discretised such that there is just one (multidimensional) Brownian increment sampled per timestep *n*, $\Delta W_{i,n}$, which we also label with the particle index *i* to indicate that dependency. Then, we define $F(X_{i,t_{k-1}}, \Delta {W}_i, \Delta \lambda _i)$ as the solution obtained at $t=t_k$ from the numerical discretisation of the SPDE using $\Delta W_i=(\Delta W_{i,1},\Delta W_{i,2},\ldots ,\Delta W_{i,N_s})$ and $\Delta \lambda _i=(\Delta \lambda _{i,1},\Delta \lambda _{i,2},\ldots ,\Delta \lambda _{i,N_s})$. We also indicate dependency of the Girsanov-adjusted negative log likelihood values via

$$ \hat{\Phi }(X_{i,t_k}, Y_{t_k}, \Delta W_i, \Delta \lambda _i). $$

The values of $\Delta W_i,\Delta \lambda _i$, $i=1,2,\ldots ,N_p$, are then updated according to Algorithm 1.

**Algorithm 1:**
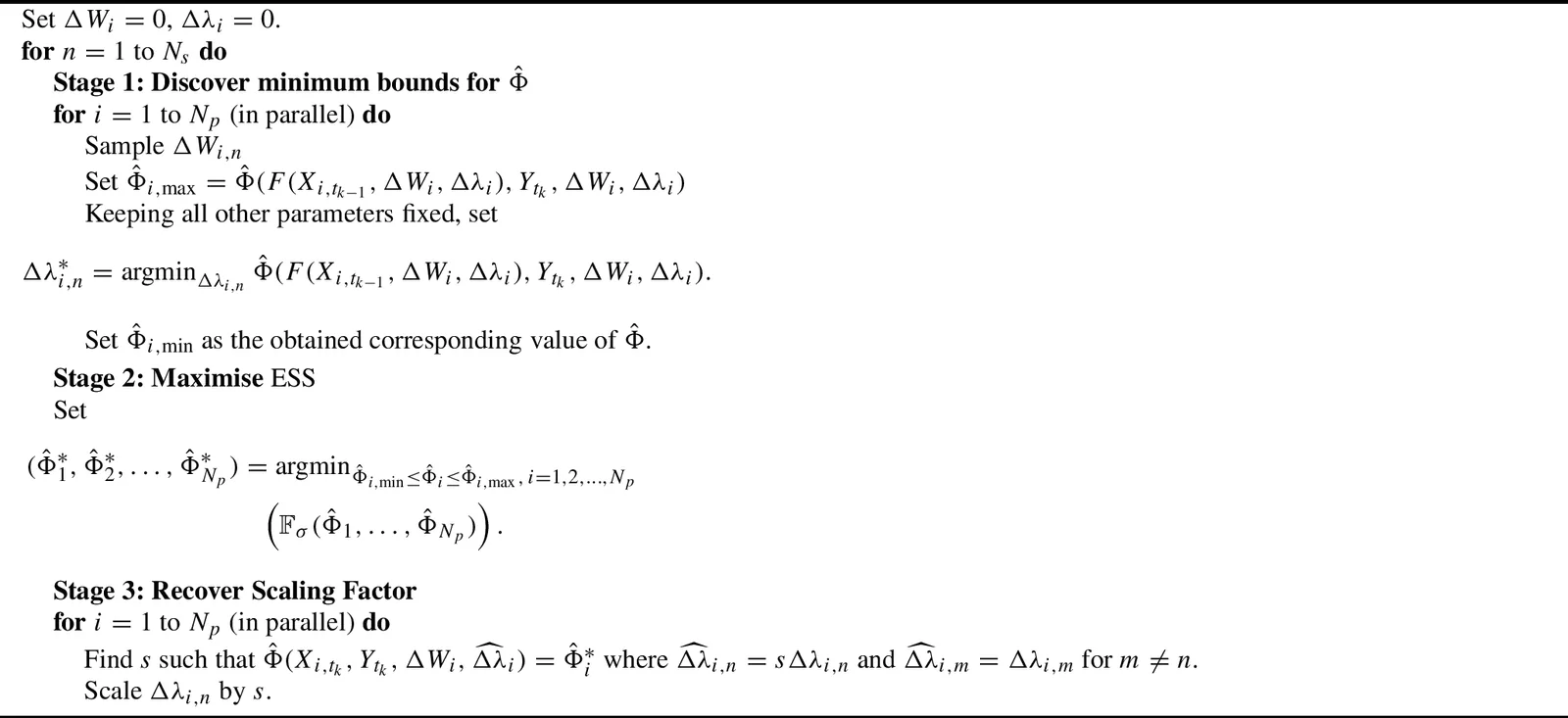
Three stage nudging

## Numerical experiments

In this section, we demonstrate the Girsanov nudging approach with some numerical experiments. These were implemented using our particle filter library (Cotter and Singh 2026) which is built around Firedrake (Ham 2023), an automated system for solving partial differential equations using the finite element method. Our library has a modular design which allows for any SPDE discretisation that is expressible in Firedrake; new discretisations are added by subclassing a model class which hands over noise realisation sampling to the particle filter so that different filter algorithms can be used seamlessly. The library makes use of Firedrake’s automated adjoint capability (Farrell et al. 2013; Mitusch et al. 2019), allowing gradients of $\hat{\Phi }$ with respect to $\Delta \lambda$ to be computed scalably and nonintrusively in the optimisation procedure. The library also makes use of message passing interface (MPI) subcommunicators so that the algorithm can be parallelised over particles as well as composed with standard spatial domain decomposition parallelism applied to the forward model. In this article, we only demonstrate the scheme with SDE and one-dimensional SPDEs, where only parallelism over particles was used, but the system is designed to be fully capable for large scale forward models, which we will consider in the future.

In our numerical experiments, we compared our particle filter with (a) the classical bootstrap particle filter, and (b) the temper-jitter particle filter of Beskos et al. (2017), Cotter et al. (2020a), Cotter et al. (2020b). This latter filter transforms from the prior distribution $\pi _{t_k^-}$ to the posterior distribution $\pi _{t_k^+}$ in a sequence of steps via intermediate distributions, $\pi _{\theta _j}$, $j=0,\ldots ,N_\theta$, with $0=\theta _0=\theta _1<\ldots <\theta _{N_\theta }:=1$, and

$$\begin{aligned} &  \frac{{{\,\textrm{d}\,}}{\pi _{\theta _j}}}{{{\,\textrm{d}\,}}\pi _{\theta _{j-1}}} = \left( L(X_{k},Y_k)\right) ^{\Delta \theta _j}, \quad \pi _{\theta _0}=\pi _{t_k^-}, \quad \\ &  \quad \Delta \theta _j = \theta _j-\theta _{j-1}. \end{aligned}$$

At each step, the temper-jitter filter selects $\Delta \theta _j$ adaptively so that the ${{\,\textrm{ESS}\,}}$ remains above some threshold (${{{\,\textrm{ESS}\,}}} > 0.8N_p$ in our experiments). The particles are weighted by the “tempered likelihood” $\left( L(X_{k},Y_k)\right) ^{\Delta \theta _j}$, and then resampled to obtain an equal weighted ensemble again. Then, “jittering” is applied, meaning that some number of Monte Carlo Markov Chain (MCMC) iterations are applied to the noise increments used to advance the particle state from $t_k$ to $t_{k-1}$, given the initial condition $X_{i,k-1}$, to obtain alternative samples from the distribution $\pi _{\theta _j}$. This has the effect of avoiding duplication of particles and improving the approximation, by selecting alternative noise increments that are statistically consistent with the target distribution. The jittering steps are applied independently in parallel for each particle and the only coupling between particles occurs in the resampling step. In our experiments we used the Preconditioned Crank Nicholson MCMC proposal of Cotter et al. (2013) in our jittering steps, in the form of their Equation (4.6) for a chosen step parameter δ>0. This process is then repeated to transform from $\pi _{\theta _k}$ to $\pi _{\theta _{k+1}}$ and so on. This process requires to continue to store the initial conditions $X_{i,k-1}$ and noise increments ${{\,\textrm{d}\,}}W_{i}$ during the resampling process.

For the experiments with the Girsanov nudging particle filter, we found that applying a small number of jittering steps (without tempering) after resampling improved the quality of the results in the case of the linear SDE example. Hence, we have done this in all of our results. In these experiments, we were mainly focused on the behaviour of the filter in terms of accuracy and stability, and we did not attempt to tune termination criteria for the optimisation solvers, leaving this for future work with more advanced versions of the filter as discussed in the summary and outlook section.

### Linear SDE

First, as a brief verification of consistency of the filter, we consider the one-dimensional linear SDE

15 $$\begin{aligned} {{{\,\textrm{d}\,}}X_t = -A X_t {{\,\textrm{d}\,}}t + D{{\,\textrm{d}\,}}W_t}, \end{aligned}$$

for $X_t\in \mathbb {R}$, with $A,D\in \mathbb {R}$ positive constants. The corresponding Girsanov-perturbed SDE is

16 $$\begin{aligned} {{{\,\textrm{d}\,}}X_t = -A X_t {{\,\textrm{d}\,}}t + D({{\,\textrm{d}\,}}W_t + \lambda (t) {{\,\textrm{d}\,}}t )}. \end{aligned}$$

We discretise (16) using the midpoint scheme,

$$\begin{aligned} (1+A \Delta t/2)X^{n+1}= &  (1 - A \Delta t/2)X^{n} + D (\Delta W^{n+1} \nonumber \\ &  + \Delta t \lambda ^{n+1}), \end{aligned}$$

where $\Delta W^{n+1}\sim N(0, \Delta t)$, and where the scheme for (15) is recovered from the choice $\lambda ^{n+1}=0$.

In our experiment, we initialise the ensemble as samples from the $N(0, D^2/(2A))$ distribution, selecting A=D=1. The observation operator is h(X)=X, and we make a noisy observation h(X)+ϵ=-0.055634 with $\epsilon \sim N(0, 0.01)$ at time t=1, with time step size Δt=1/10. For this problem we are able to compute the exact posterior distribution using a Kalman filter formula; this allows us to compute errors and compare methods.

The Girsanov nudging particle filter was used with 5 jittering steps with MCMC step parameter δ=0.05 after resampling. The Stage 1 optimisation was solved using L-BFGS (Limited memory version of the Broyden-Fletcher-Goldfarb-Shanno algorithm) (Nocedal and Wright 2006), using the SciPy (Virtanen et al. 2020) implementation as wrapped in the pyadjoint library (Mitusch et al. 2019) with parallelism over the particles. The Stage 2 optimisation was solved using the L-BFGS-B (bounded version of L-BFGS) provided by SciPy, and the Stage 3 optimisation was solved using Brent’s method (Brent 2013) also provided by SciPy, also with parallelism over the particles. Note that, the parameter σ in (14) is taken as 0.1 in this experiment.

We compared the Girsanov nudging filter with the bootstrap filter and the temper-jitter filter, the latter of which used MCMC step parameter δ=0.15 and 5 jittering steps after each tempered resampling step.

We compare the mean and the variance of the posterior distribution with the exact values for the undiscretised SDE (hence the filter results will contain timestepping errors). Results are presented in Tables 1 and 2; we conclude that the nudging particle filter produces estimates that are of similar accuracy to the temper-jitter filter. Remaining errors appear to be due to the time discretisation of the forward model.

**Table 1:** Filter estimates of the posterior mean for the linear SDE experiment, averaged over 500 independent runs with different random seeds. Estimator error is computed as $|\text {true} - \text {estimated}|$. In preliminary single-run experiments, the effective sample size (ESS) ratio for the bootstrap filter was approximately 15–20% and for the nudging filter was approximately 50–60%, demonstrating that nudging raises the ESS relative to the bootstrap filter. ESS is not reported for the temper-jitter filter since the tempering is adapted to ensure ESS $\approx 80\%$ at each tempering step

Filter	Ensemble size	Mean of estimators	Estimator error
*True mean:* -0.05454311			
Bootstrap	75	−0.05551755	0.00097444
	150	−0.05434665	0.00019646
	300	−0.05442847	0.00011464
Temper−Jitter	75	−0.05494858	0.00040547
	150	−0.05471432	0.00017121
	300	−0.05457935	0.00003624
Nudge(+Jitter)	75	−0.05410795	0.00043516
	150	−0.05432655	0.00021656
	300	−0.05474613	0.00020302

**Table 2:** Filter estimates of the posterior variance for the linear SDE experiment, averaged over 500 independent runs with different random seeds. Estimator error is computed as $|\text {true} - \text {estimated}|$. In preliminary single-run experiments, the effective sample size (ESS) ratio for the bootstrap filter was approximately 15–20% and for the nudging filter was approximately 50–60%, demonstrating that nudging raises the ESS relative to the bootstrap filter. ESS is not reported for the temper-jitter filter since the tempering is adapted to ensure ESS $\approx 80\%$ at each tempering step

Filter	Ensemble size	Mean of estimators	Estimator error
*True variance:* 0.00980392			
Bootstrap	75	0.00985380	0.00004988
	150	0.00977820	0.00002572
	300	0.00981366	0.00000974
Temper-Jitter	75	0.00965919	0.00014473
	150	0.00976763	0.00003629
	300	0.00980892	0.00000500
Nudge(+Jitter)	75	0.00988255	0.00007863
	150	0.00986034	0.00005642
	300	0.00983085	0.00002693

### Stochastic Kuramoto-Sivashinsky equation

To investigate the stability and accuracy of the Girsanov nudging approach, we performed data assimilation experiments using the stochastic Kuramoto-Sivashinsky (SKS) equation with additive noise (Duan and Ervin 2001),

17 $$\begin{aligned} {{\,\textrm{d}\,}}u + \left( \alpha u_{xxxx} + \beta u_{xx} + \gamma uu_x\right) {{\,\textrm{d}\,}}t = c {{\,\textrm{d}\,}}W, \end{aligned}$$

solved on the interval [0, *L*] with periodic boundary conditions u(L,t)=u(0,t), where α, β, γ and *c* are constants, and where *W* is a spacetime white noise (cylindrical in space and Ito in time). In the absence of noise, this equation has a global attractor and exhibits chaotic behaviour for suitable parameter choices (Papageorgiou and Smyrlis 1991). The stochastic extension has random attractors with stable long time behaviour (Yang 2006). This makes the SKS model very useful as a tool for rapidly benchmarking particle filters.

We discretised (17) using the second degree continuous Lagrangian finite element space $V_h$ on a regular mesh with $N_v$ vertices and mesh width $h=L/N_v$, using a $\mathcal {C}^0$ interior penalty treatment of the fourth order term (Brenner and Li 2005). For the time discretisation, we consider the mid-point scheme on a uniform time mesh. The spatially discrete continuous time scheme is given by

18 $$\begin{aligned} &  \left( {{\,\textrm{d}\,}}u_h,v_h\right) - \left( \beta u_{h,x}{{\,\textrm{d}\,}}t, v_{h,x}\right) + a\left( \alpha u_{h}{{\,\textrm{d}\,}}t, v_h\right) \nonumber \\ &  \quad -\left( \dfrac{\gamma }{2} \left( u_{h}\right) ^{2}{{\,\textrm{d}\,}}t, v_{h,x}\right) =c{{\,\textrm{d}\,}}W_h[v_h], \quad \forall v_h \in V_h, \end{aligned}$$

where

$$\begin{aligned} a(u,v)&= (u_{xx},v_{xx}) + \langle \{u_{xx}\}, [v_x]\rangle + \langle \{v_{xx}\}, [u_x]\rangle \\&\quad + \dfrac{\eta }{h}\langle [u_x], [v_x]\rangle , \\ (u,v)&= \int _0^L uv{{\,\textrm{d}\,}}x, \\ \langle u,v \rangle&= \sum _{i=1}^{N_v}u(z_i)v(z_i),\\ \{u\}|_{z_i}&= (u(z_i^+)+u(z_i^-))/2, \\ {[}u]|_{z_i}&= u(z_i^+)-u(z_i^-), \end{aligned}$$

where $^+$ and $^-$ indicate right and left limiting values respectively, η is the (mesh independent) interior penalty parameter, and

19 $$\begin{aligned} {{\,\textrm{d}\,}}W_h[v] = \frac{1}{h^{1/2}}{\sum _{i=1}^{N_v}} \left( \int _{ih}^{(i+1)h} v {{\,\textrm{d}\,}}x\right) {{\,\textrm{d}\,}}W_i, \end{aligned}$$

with $\{W_i\}_{i=1}^{N_v}$ a set of $\textrm{iid}$ Brownian motions, so that

20 $$\begin{aligned}&\mathbb {E}\left( \left( \int _{t_0}^{t_1}{{\,\textrm{d}\,}}W_h[v]\right) \left( \int _{t_0}^{t_1}{{\,\textrm{d}\,}}W_h[w]\right) \right) \nonumber \\&\quad = \int _{t_0}^{t_1}\frac{1}{h}{\sum _{i=1}^{N_v}} \left( \int _{ih}^{(i+1)h} v {{\,\textrm{d}\,}}x\right) \left( \int _{ih}^{(i+1)h} w {{\,\textrm{d}\,}}x\right) {{\,\textrm{d}\,}}t, \nonumber \\&\quad \approx \int _{t_0}^{t_1}\int _0^L vw {{\,\textrm{d}\,}}x {{\,\textrm{d}\,}}t. \end{aligned}$$

We apply the Girsanov perturbation after discretising in space but before discretising in time, replacing ${{\,\textrm{d}\,}}W_i\mapsto {{\,\textrm{d}\,}}W_i + \lambda _i(t){{\,\textrm{d}\,}}t$, $i=1,2,\ldots ,N_v$ in (19). After applying the implicit midpoint rule, the fully discretised Girsanov perturbed system is

21 $$\begin{aligned}&\left( {u_h^{n+1}-u_h^n},v_h\right) - \left( \beta \Delta tu_{h,x}^{n+1/2}, v_{h,x}\right) \nonumber \\&\quad + a\left( \Delta t \alpha u_{h}^{n+1/2}, v_h\right) \nonumber \\&\quad -\left( \dfrac{\gamma \Delta t}{2} \left( u_{h}^{n+1/2}\right) ^{2}, v_{h,x}\right) =c\widehat{\Delta W_h}^n[v_h], \quad \forall v_h\in V_h, \end{aligned}$$

where

$$\begin{aligned} \widehat{\Delta W_h}^n[v] = \frac{1}{h^{1/2}}{\sum _{i=1}^{N_v}} \left( \int _{ih}^{(i+1)h} v {{\,\textrm{d}\,}}x\right) \left( \Delta W_i^n + \lambda _i^n \Delta t\right) , \end{aligned}$$

where $\Delta W_i^n$ are N(0,Δt) random variables. Here we see that λ takes the role of a piecewise constant function in space. The unperturbed discretised SPDE is recovered for $\lambda _i^n=0$.

We employed a 90-particle ensemble in our numerical particle filtering studies applied to the SKS model. This is motivated by common ensemble sizes of data assimilation and ensemble uncertainty quantification systems for operational weather forecasting, where the high computational cost of the forward models limits ensemble sizes; our goal is to obtain stable particle filter configurations in the low ensemble size setting.

In the numerical setup of the SKS model, we chose the length of the spatial domain L=4 with 100 cells (so that there are 200 degrees of freedom, since the finite element space is piecewise quadratic), and we take the diffusion coefficient α=0.03, the anti-diffusion term β=1.1, the advection parameter γ=1, and the noise parameter c=2.5. We used η=5 for the interior penalty parameter.

For all the numerical experiments, the initialisation of particles and reference trajectory (“truth”) is constructed in the following way. First, we run the above numerical simulation of the SKS model over 200 steps with the following initial condition,

$$\begin{aligned} u_{in}(x)= &  \left( \dfrac{0.4}{e^{(10x-403./15.)} + e^{(-10x+403./15.)}} \right. \\ &  \left. + \dfrac{1}{e^{(10x-203./15.)}+e^{(-10x+203./15.)}}\right) . \end{aligned}$$

**Fig. 1:**
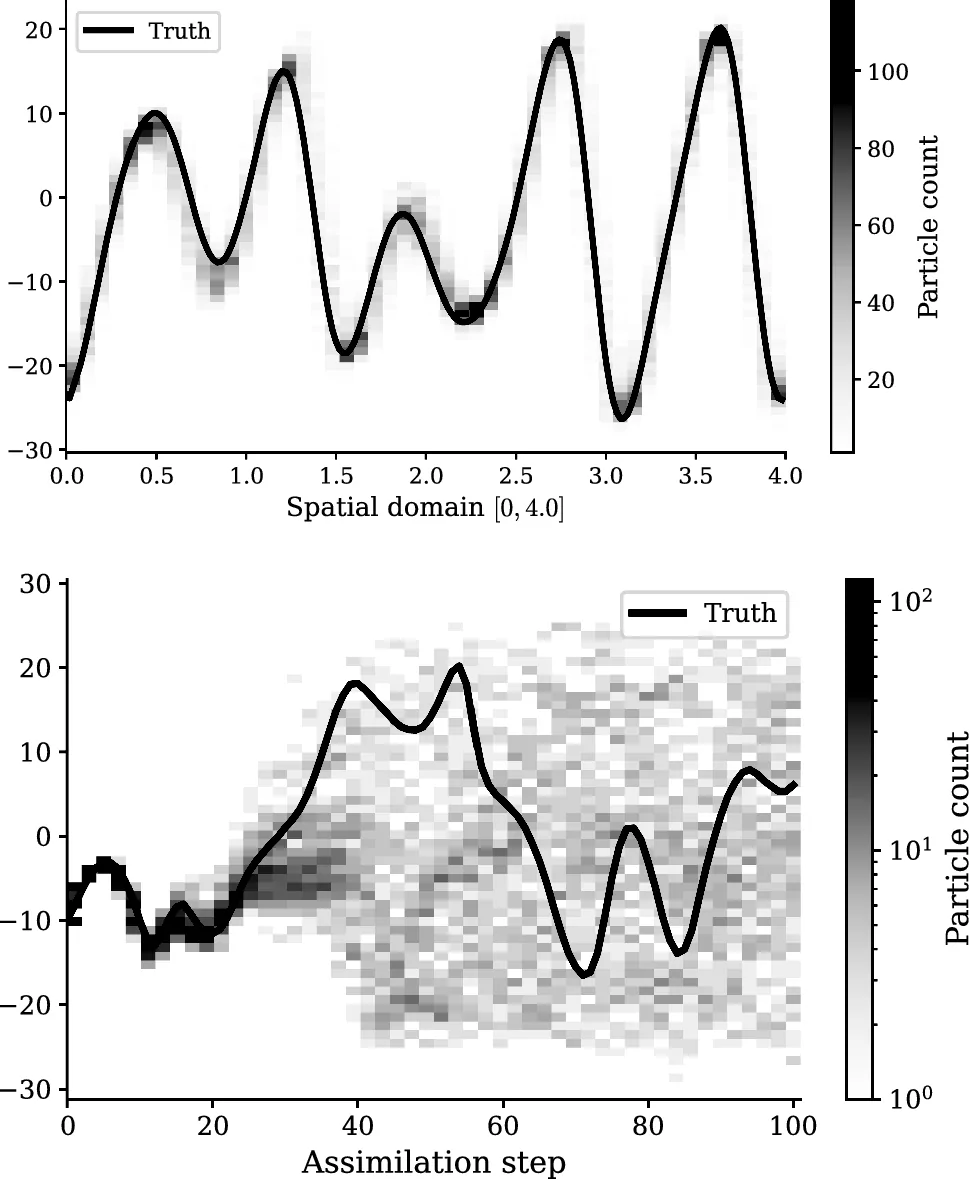
Top: Prior ensemble distribution at t=0 before data assimilation. The histogram shows the initial spread of all particles drawn from the prior distribution. The black curve represents the Kuramoto-Sivashinsky system’s “true” initial conditions. The initial ensemble spread represents the prior uncertainty in the state estimate. Bottom: Evolution of ensemble without data assimilation showing that ensemble spread grows and truth is no longer tracked

Then, the solution at the final time of the above simulation is considered as the initial condition $u_{0}$ for the truth and all the particles. Figure 1 displays the initialisation of truth and particles. We observe that the particle distribution is somewhat spread around the truth. Furthermore, we have demonstrated evolution of particles, without data assimilation. As expected, a significant spread is observable when comparing these particles to the truth.

We took measurements at 10 equispaced points in the interval [0, 4]. All observation processes are perturbations of the “true” trajectory with $\textrm{iid}$ measurement errors of distribution $\mathcal {N}(0, 2.5)$. The observations are taken every 5 timesteps. In the second stage optimisation, the parameter σ=0.01 in (14) is considered.

To quantify the behaviour of the particle filters, we consider the following widely used metrics; Root mean-square error (RMSE), relative bias (RB) and root ensemble spread (RES), defined by

22 $$\begin{aligned}&\text{ RMSE } := \dfrac{1}{N_p} \sum _{i=1}^{N_p}\dfrac{\Vert {y_{\textrm{true}}} - h(X_i)\Vert _{\ell ^2}}{\Vert {y_{\textrm{true}}}\Vert _{\ell ^2}}, \end{aligned}$$

23 $$\begin{aligned}&\text{ RB } := \dfrac{\Vert {y_{\textrm{true}}} - h(\bar{X})\Vert _{\ell ^1}}{\Vert {y_{\textrm{true}}}\Vert _{\ell ^1}}, \qquad \text{ where } h(\bar{X}) = \frac{1}{N_p}\sum _{i=1}^{N_p}h(X_i), \end{aligned}$$

24 $$\begin{aligned}&\text{ RES } := \dfrac{1}{N_p - 1} \sum _{i=1}^{N_p}\dfrac{\Vert h(\bar{X})-h(X_i)\Vert _{\ell ^2}}{\Vert {y_{\textrm{true}}}\Vert _{\ell ^2}}. \end{aligned}$$

Here ${y_{\textrm{true}}} = h(X^{\dagger })$ denotes the observation operator applied to the reference trajectory $X^{\dagger }$, and the $\ell ^1$, $\ell ^2$ norms are taken over the *M* observation points.

**Fig. 2:**
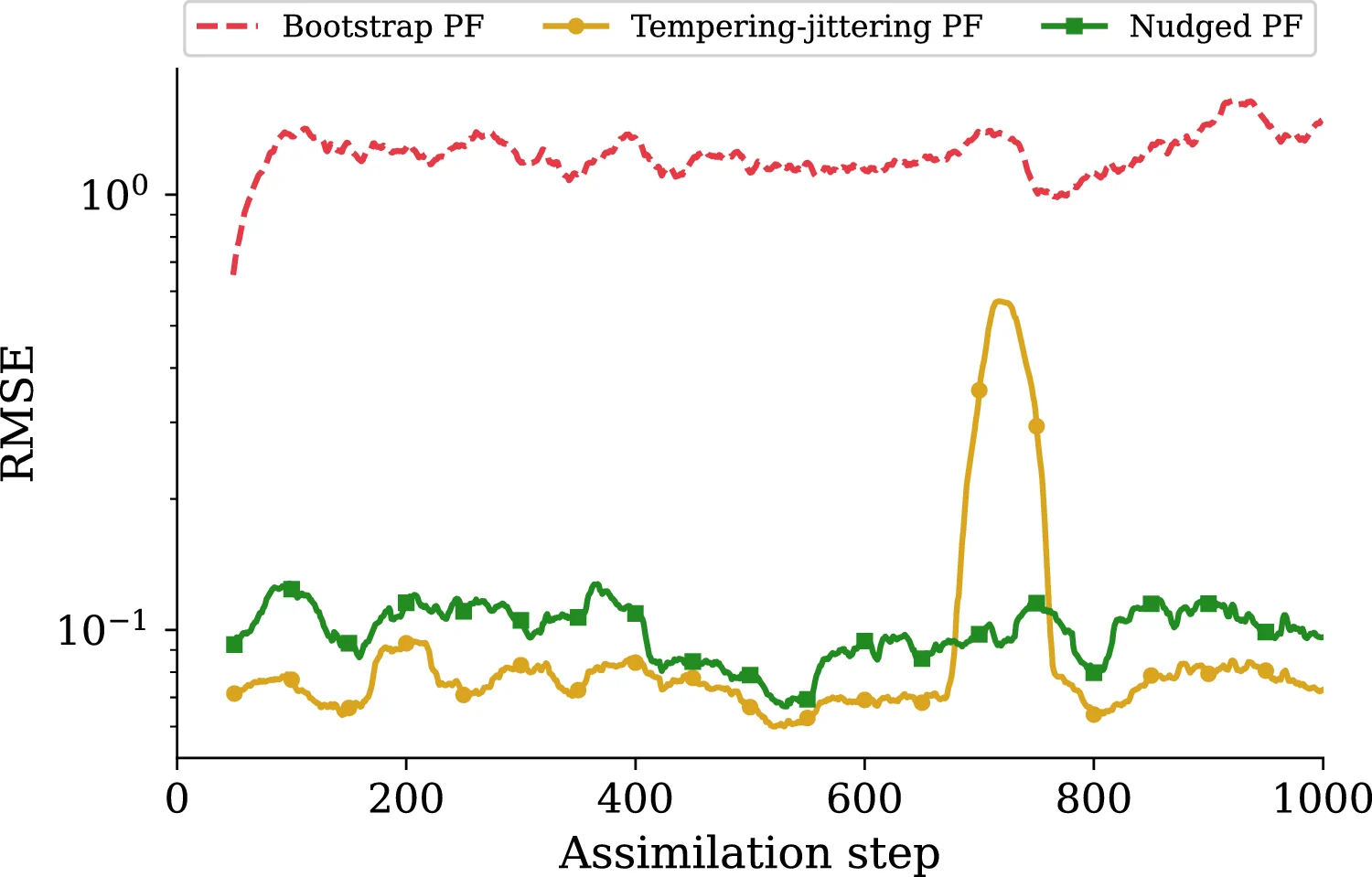
RMSE plots for the bootstrap, temper-jitter and Girsanov nudged filters applied to the SKS equation

**Fig. 3:**
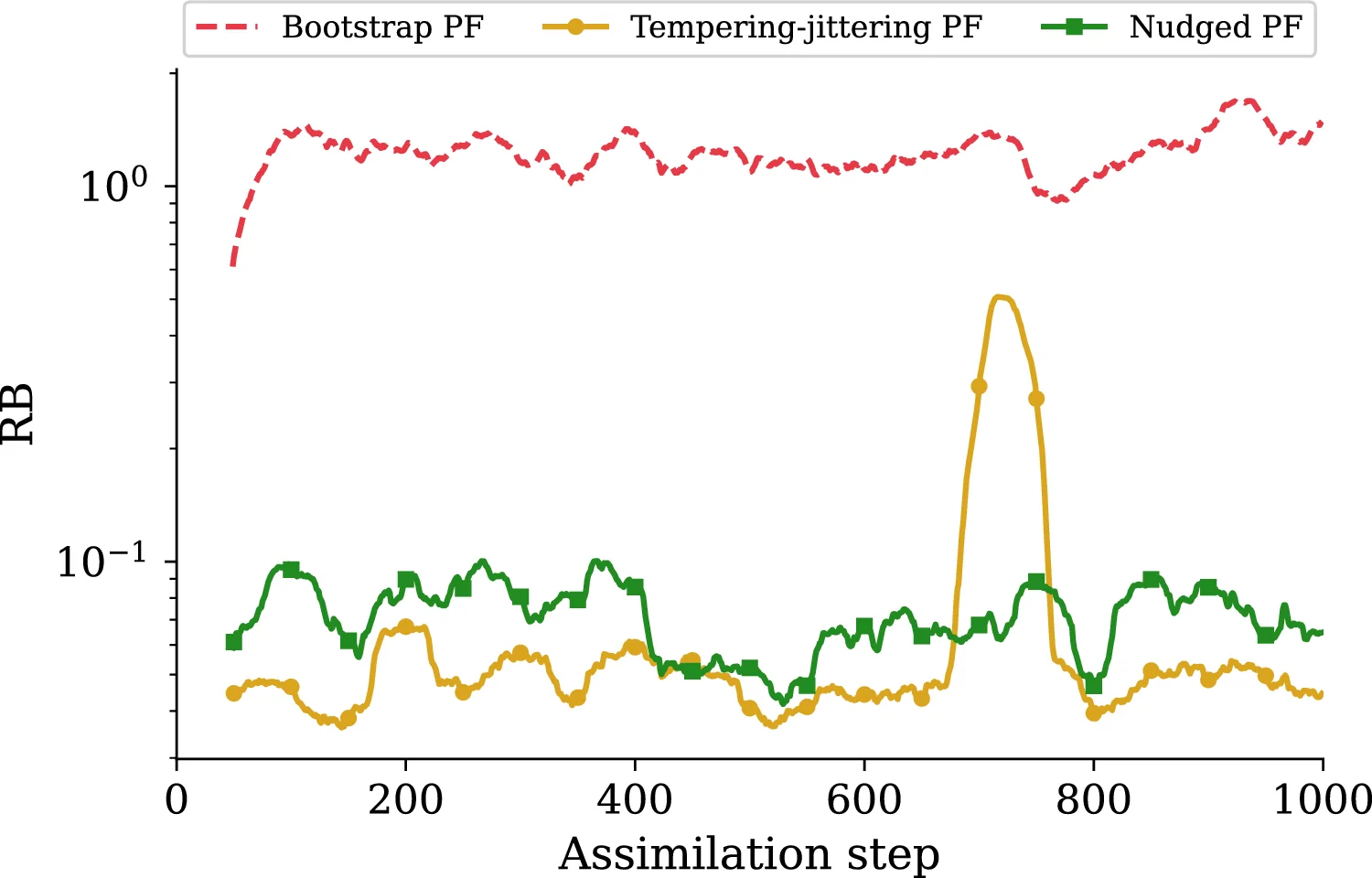
RB plots for the bootstrap, temper-jitter and Girsanov nudged filters applied to the SKS equation

**Fig. 4:**
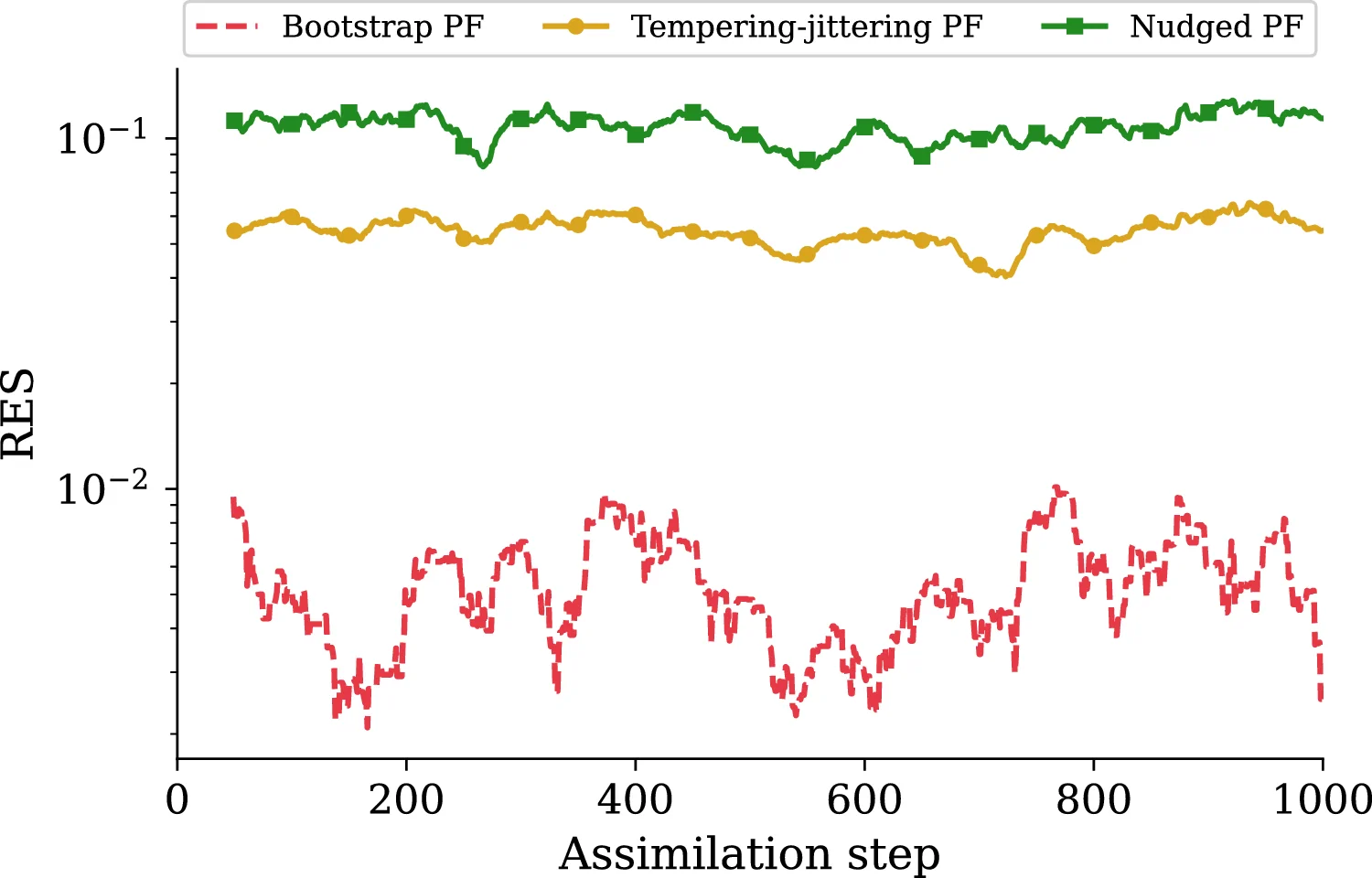
RES plots for the bootstrap, temper-jitter and Girsanov nudged filters applied to the SKS equation

**Fig. 5:**
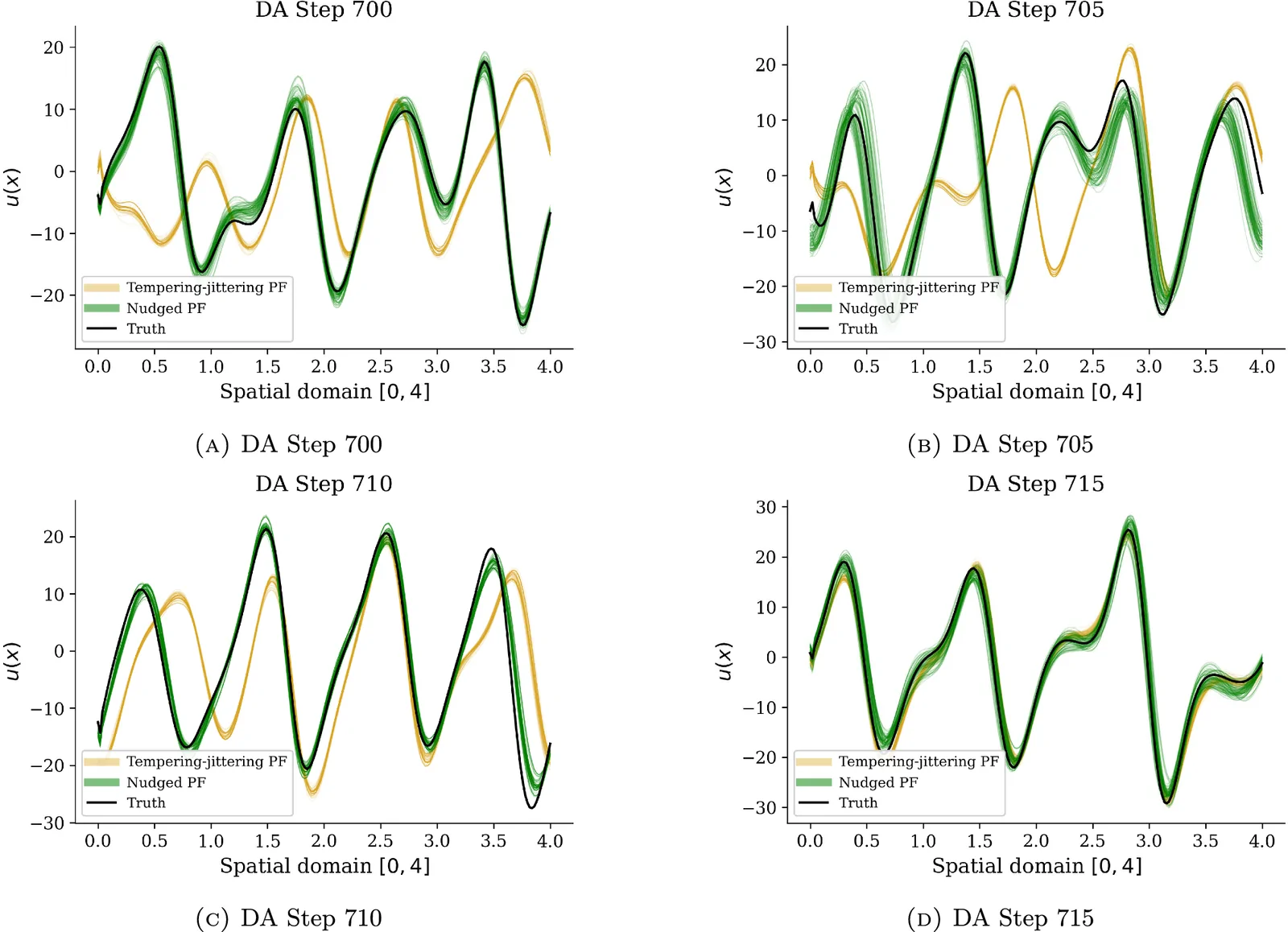
Comparison of ensemble vs truth for temper-jitter PF vs Nudged PF for various data assimilation step

Time series for these metrics are shown in Figs. 2, 3 and 4. We observe that both the temper-jitter and Girsanov nudged particle filters are able to track the true solution in a stable manner, whilst the bootstrap filter error is saturating ie no better than just running the forward model without assimilating data. We also observe that the Girsanov nudged particle filter is producing an ensemble which is more spread than the temper-jitter filter. This appears to be because the former filter moves the particles in regions of highest likelihood to regions of lower likelihood to try to balance out the weights of the ensemble, whilst the latter filter repeatedly resamples during the tempering procedure to produce an ensemble where most of the particles originate from the same state at the previous assimilation step. However, we also observe that the temper-jitter filter undergoes an excursion at around 700 data assimilation steps, where the ensemble is drifting away from the truth; at later times this excursion is arrested and the relative bias decreases again. This appears to occur because of an “extreme event” in the true dynamics, leading to a solution that is unlikely with respect to the filtering distribution before this point, perhaps due to the temporary creation or destruction of a bump in the solution. We do not observe this large excursion with the Girsanov nudged filter, suggesting that it was able to move the ensemble closer to the region of high likelihood using the control variables. We see some evidence for this in Fig. 5, which shows that the true solution undergoes a merger of two bumps into one, which does not occur in any of the temper-jitter states. The Girsanov nudged filter ensemble particles all undergo this merge after a shorter time when the true solution is outside the spread of the ensemble in that region of the domain.

**Fig. 6:**
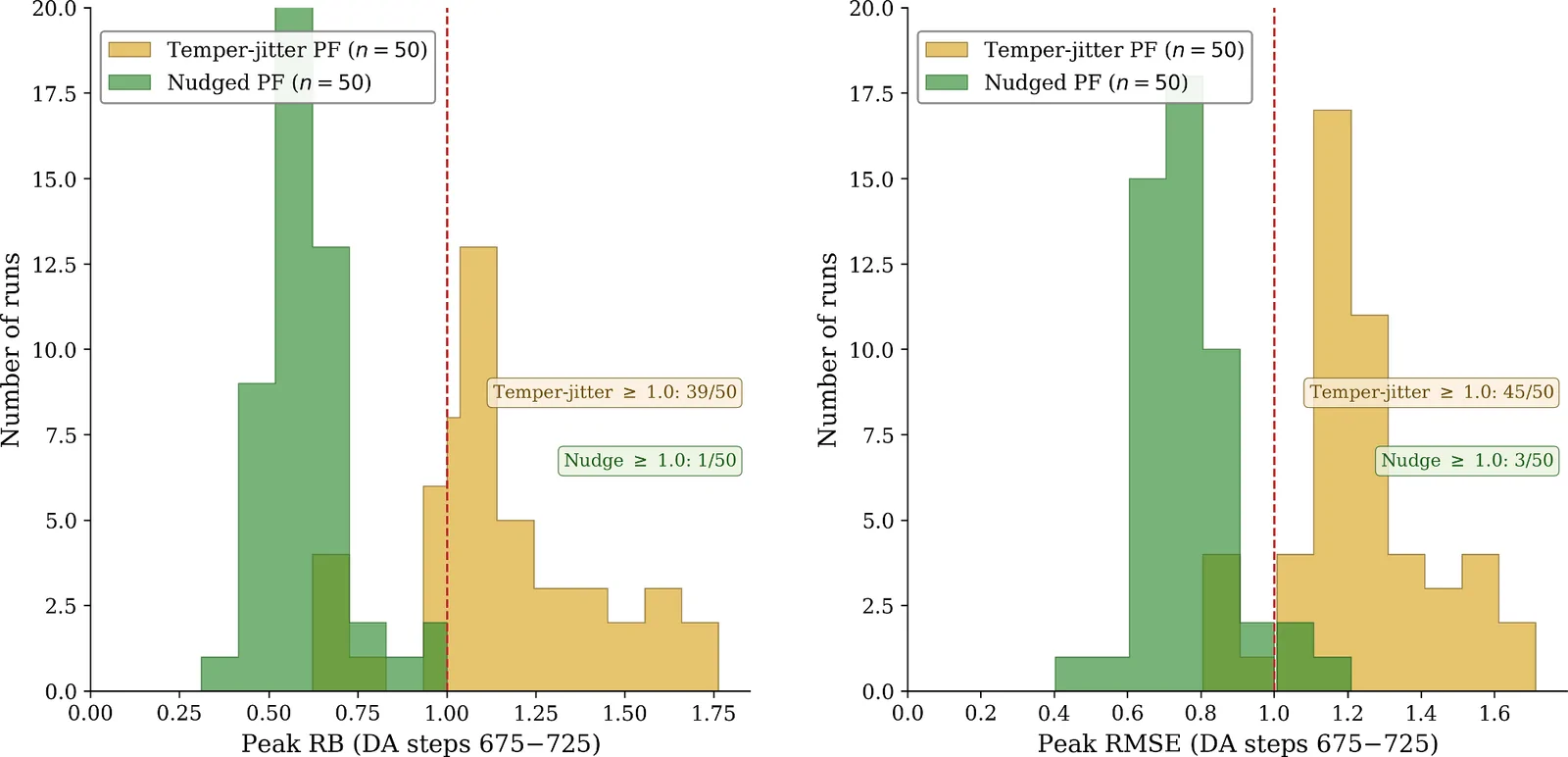
Peak RB (left) and RMSE (right) over the excursion window(DA step 675-725), computed from 50 independent runs with different random seeds

We investigated the robustness of the excursion behaviour by restarting both filters from saved ensemble checkpoints at DA step 675, running each forward for 50 assimilation steps with 50 different random seeds for the model noise and resampling algorithm. Figure 6 shows the peak RB and RMSE attained during this window. The temper-jitter filter exceeds $\text{ RB } \ge 1.0$ in 39 out of 50 runs, whereas the nudging filter does so in only 1 out of 50 runs. Similar behaviours follow for $\text{ RMSE }$.

**Fig. 7:**
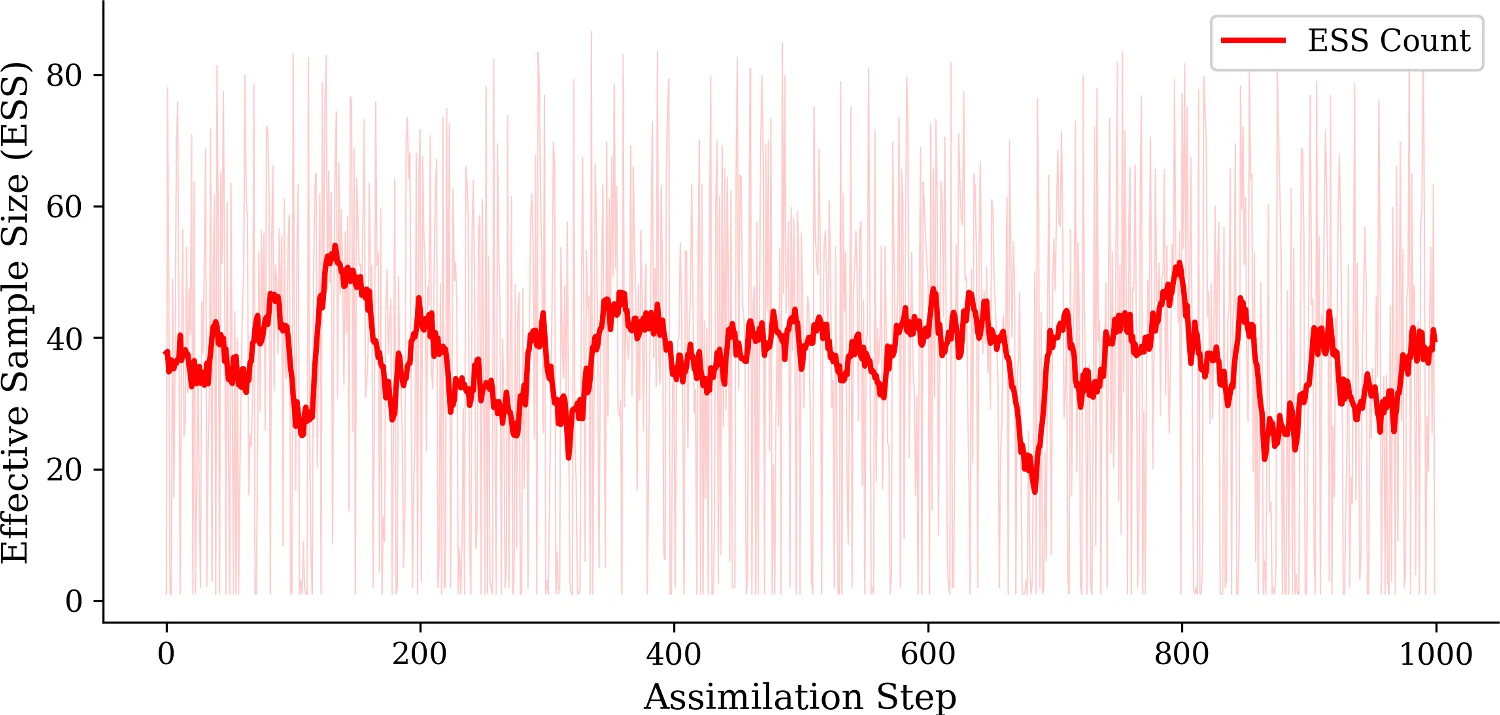
${{\,\textrm{ESS}\,}}$ for the Girsanov nudged filter

Figure 7 shows the time series for ${{\,\textrm{ESS}\,}}$ for the Girsanov nudged filter during the experiment. We see that it fluctuates a lot but it remains a significant fraction of the total ensemble size 90 throughout, with the lowest value during the extreme event studied in Fig. 5, as might be expected.

**Fig. 8:**
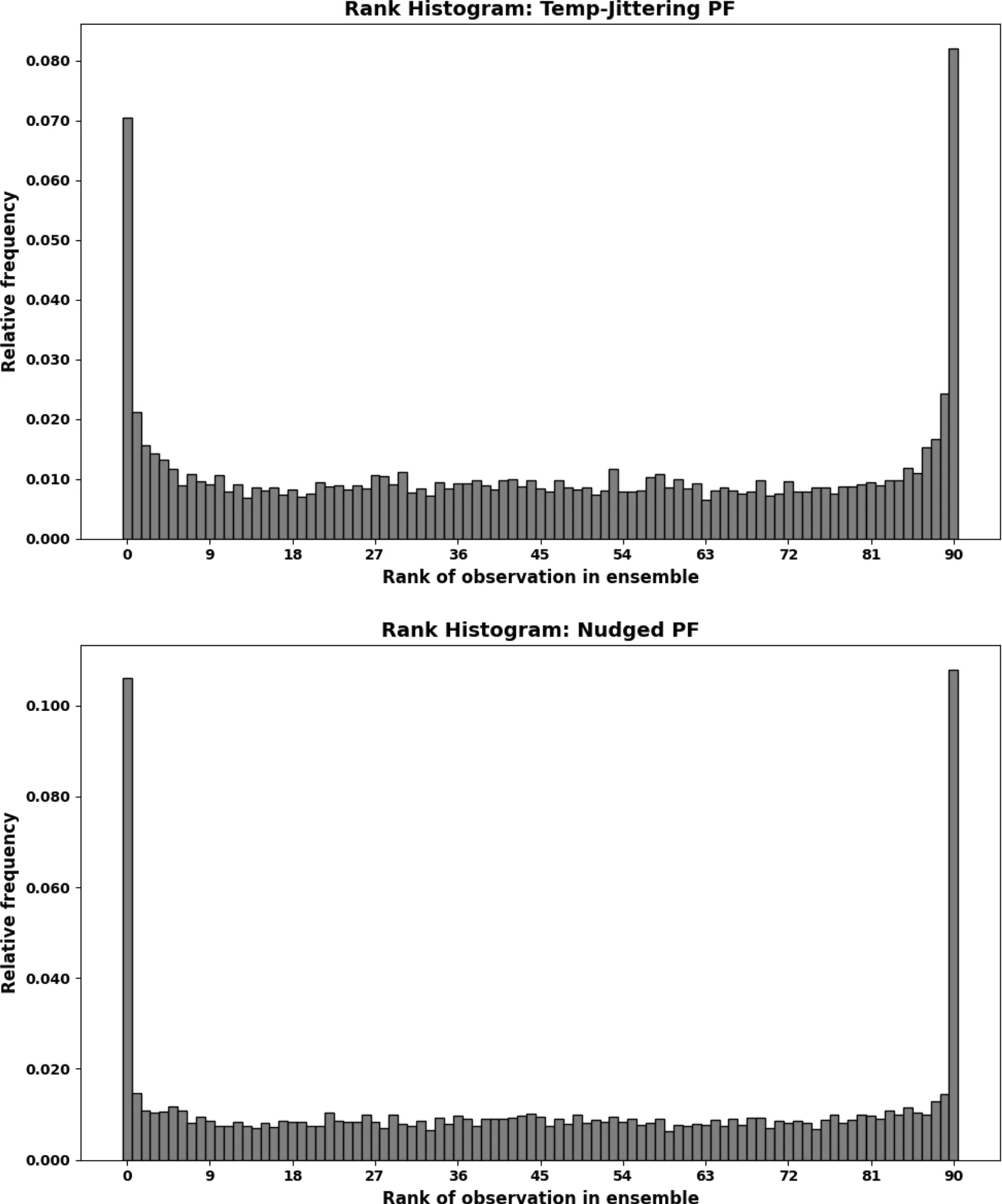
Comparison of rank histograms. Top: the temper-jitter filter. Bottom: the Girsanov nudging filter

As a final comparison, we examine rank histograms for the particle filters. These are computed following the procedure described in e.g. (Reich and Cotter 2015) (Section 4.4). As expected, the bootstrap filter produces extremely poor results and is not shown. We observe that both the temper-jitter and Girsanov nudging filters behave quite similarly, with flat interiors but significant spikes at the sides of the histogram which are probably due to the excursions where the true value moves outside the ensemble Fig 8.

#### Remark 1

*(Computational complexity)* The computational cost of Algorithm 1 is dominated by Stage 1, which requires $N_p$ forward and adjoint PDE solves per substep. These steps are inherent to any adjoint-based proposal method and are *embarrassingly parallel* across particles. In our experiments with $N_p = 90$ particles and $N_s = 5$ substeps, Stage 1 typically accounts for 72–96% of the runtime per assimilation cycle. Stage 2, which optimises over $N_p$ particles, requires 4–7% of the runtime with L-BFGS-B converging in $\mathcal {O}(10)$ iterations. Stage 3 contributes 0–20% depending on how many particles require scaling adjustments; when most particles achieve optimal cost at the minimum bound, Stage 3 is essentially free. The total runtime per assimilation step is approximately 75–85 s on a dual-socket Intel Xeon Silver 4215 workstation (16 physical cores, 2.5 GHz). However, we have not attempted to tune the tolerances for these stages, so we are probably doing more computation than necessary. In these experiments, our goal was mainly to explore the stability and accuracy of the global nudging approach and we will investigate more efficient implementations in future work.

## Summary and outlook

We presented a nudging approach to particle filters for SPDEs, based on introducing a Girsanov perturbed model. This allows the choice of control variables which can be used to steer the ensemble towards regions of higher likelihood. We propose that the control variables need to be optimised collectively in order to avoid low ESS and consequent filter divergence. We introduced a formulation of an optimisation problem that allows the control variables to be solved in three stages; these three stages separate the nonlinearity of the ESS formula and the nonlinearity of the forward model, and allow computations in the control variable space to be made in parallel across the ensemble of particles. We presented numerical results that demonstrate the behaviour of the filter, using our parallelised implementation developed using Firedrake, making specific use of its automated adjoint capabilities for gradient based optimisation. In our comparisons, we used the temper-jitter filter as a scalable reference, since it has been rigorously analysed and tested on numerous SDE and SPDEs. We observed that for the same ensemble size of 90, the Girsanov nudging filter showed a larger ensemble spread than the temper-jitter filter, with a larger relative bias in general. However, the Girsanov filter showed signs of additional stability, recovering much more quickly after an extreme event when the true solution became unlikely with respect to the filtering distribution. This demonstrates the potential of Girsanov nudging for building more robust particle filters for PDEs.

It is clear that further work is needed to develop the filter, so that higher values of ESS can be achieved. We plan to develop extensions of the filter that interleave nudging with tempering and jittering, in the hope of combining the stability of one filter with the accuracy of the other. More engineering work is also needed to tune optimisation parameters in order to avoid wasting iterations (each of which involves solving the forward model) when they do not contribute to the stability or accuracy of the filter. We also plan to apply these particle filters to larger scale SPDEs using high performance computing facilities, bringing our work closer to applications.

## Data Availability

No datasets were generated or analysed during the current study.
